# Social capital, population health, and the gendered statistics of cardiovascular and all-cause mortality

**DOI:** 10.1016/j.ssmph.2021.100971

**Published:** 2021-11-19

**Authors:** Dragos Simandan

**Affiliations:** Faculty of Social Sciences, Brock University, 1812 Sir Isaac Brock Way, St. Catharines, Ontario, Canada, L2S 3A1

**Keywords:** Community, Social capital, Mortality, Population health, Gender, Ideology, Trust, Positionality

## Abstract

Scholars in the field of population health need to be on the constant lookout for the danger that their tacit ideological commitments translate into systematic biases in how they interpret their empirical results. This contribution illustrates this problematic by critically interrogating a set of concepts such as tradition, trust, social capital, community, or gender, that are routinely used in population health research even though they carry a barely acknowledged political and ideological load. Alongside this wider deconstruction of loaded concepts, I engage critically but constructively with Martin Lindström et al.'s paper “Social capital, the miniaturization of community, traditionalism and mortality: A population-based prospective cohort study in southern Sweden” to evaluate the extent to which it fits with other empirical findings in the extant literature. Taking as a point of departure the intriguing finding that social capital predicts cardiovascular and all-cause mortality only for men, but not for women, I argue that future research on the nexus of social capital, health, and mortality needs to frame gender not only as a demographic and statistical variable, but also as an ontological conundrum and as an epistemological sensibility.

## 1 Introduction

The journal *SSM - Population Health* is an excellent opportunity for practicing interdisciplinarity and transdisciplinarity to the extent that its contributors represent a variety of disciplines within the social sciences and the biomedical sciences, and the peer-review system prioritizes topical expertise rather than a particular disciplinary affiliation. This opportunity translates into clashes between authors and peer-reviewers ad well as between peer-reviewers of the same paper regarding conceptual, theoretical, and methodological commitments on how to approach a given empirical problem. I had a chance to experience this ideological clash as a reviewer of Lindström et al.'s fascinating paper “Social capital, the miniaturization of community, traditionalism and mortality: A population-based prospective cohort study in southern Sweden”. In this commentary I aim to describe this ideological clash because it strikes me as a good way to collectively learn how to negotiate pluralism and difference in the social study of health (cf. (Simandan, Rinner, & Capurri), in press), but also as a good way to make sense of what Lindström et al.'s empirical findings say and don't say.

## 2 Ideologies of social capital and population health research

Lindström et al.'s paper is a prospective cohort study exploring the associations between social capital and mortality using the 2008 public health survey in Scania and linking it to the regional prospective public death register data. It is part of a broader research effort to evaluate the extent to which a popular social science construct – social capital – can predict important outcomes for population health and wellbeing. The fact that the paper is a significant contribution to this field becomes especially apparent when juxtaposed with the systematic review of prospective studies on this topic by Choi et al., 2014, which found that the pooled estimates of the fourteen studies under consideration “showed no association between most social capital dimensions and all-cause mortality, CVD or cancer” (p. 1895) and warned that “lack of consensus on measurements for social capital hinders the comparability of studies and weakens the evidence base” (p. 1895). Interestingly, of the seven dimensions of social capital identified by Choi et al. - social participation, social network, civic participation, social support, trust, norm of reciprocity and sense of community – only two dimensions – social participation and civic participation – appeared to have some predictive value for mortality, but even this finding was limited to cases of “comparing the most extreme risk comparisons” (p. 1895). After systematic reviews with such meagre results, one would expect some generalized despondency among researchers in this area and a strong temptation to move on to other, more promising research topics. To their credit, the coauthors have shown dogged persistence on this problem, continuing to research the association between social capital and mortality even after the publication of Choi et al., 2014 (e.g. Lindström & Rosvall, 2019). However, scholars like myself who tend to approach the social study of health partly through the lens of critical theory (Simandan, 2010, 2011, Simandan, 2017a, Simandan, 2017b, Simandan, 2017c, 2018) are compelled to look at that dogged persistence as a clue to an unacknowledged ideological commitment. Seen in this light, “social capital” is a concept that carries a tacit ideological load as an articulation of fuzzier values and beliefs that promote what Miranda Joseph beautifully called “the romance of the community” (Joseph, 2002). It embodies in a convenient and “catchy” phrase pre-analytical intuitions that prosocial behavior, social participation, civic participation, generalized trust, caring, solidarity, and an “other” orientation are unadulterated goods that should be promoted in public discourse and academic discourse. Of course, what this warm and cozy feeling toward the concept of “social capital” leaves out is the dark side of community dynamics, represented by ostracism, social exclusion, marginalization, persecution, “us” versus “them” polarized thinking, and so on. To be sure, the revised version of Lindström et al.'s article makes a gesture toward acknowledging the literature on this darker side (e.g. Pawar, 2006; Villalonga-Olives & Kawachi, 2017), but is that enough? I don't think so. In everyday life as well as in academic discourse we usually convince ourselves and others that a particular issue is good or desirable, by showing how the presence of the good thing triggers other good things in its wake, whereas its absence brings negative outcomes. This maneuver can be argued to underpin most of the research on social capital and health outcomes: in our heart of hearts, we want to prove that more social capital leads to better health and reduced mortality and if reality refuses to cooperate with us, we need to figure out more ingenious study designs that yield the results we want to get. In other words, we need to be on the constant lookout for the danger that our tacit ideological commitments translate into systematic biases in the manner in which we interpret our empirical results. In the discussion section of his paper, Lindström et al. write that their new findings “partly contrast” with the deflating conclusions of the systematic analysis by Choi et al., 2014. That particular choice of framing is telling because it pushes in the background the equally valid framing of saying that their findings “largely agree” with Choi et al.‘s overall conclusion. Let's recapitulate the actual results. Social capital fails to predict cancer and other causes mortality for both men and women. The finding of an association between social capital and cardiovascular and all cause mortality, on the other hand, holds only for men, but not for women. That is alarming because we do not have a readily available, widely accepted, and empirically confirmed biomedical or psychosocial explanation for this gendered outcome. Lindström et al. refuse to see the elephant in the room and move past the intriguing finding of a gendered disparity without any attempt at either explaining it or looking into the available literature to try to find plausible explanations. An earlier population-based prospective study from Finland that operationalized individual-level social capital into three factors (leisure participation, interpersonal trust, and residential stability) found that the first two factors did predict both all-cause and cardiovascular mortality in women, specifically (Hyyppä et al., 2007). This should give us pause. The million dollar question arising from Lindström et al. (2021) is whether the gendered disparity is ontological (a real objective finding that social capital does not predict mortality for women) or methodological (an artifact of what philosophers of statistics ascribe, variously, to statistical paradoxes, “p-hacking”, or insufficient effort to meet the “severity criterion” in the testing of hypotheses, cf. Head et al., 2015; Mayo, 2018; Wainer & Brown, 2004). In my estimation, even though there is some prior literature suggesting that the impact of social capital on health may be differentiated by gender (e.g. Hyyppä et al., 2007; Karhina et al., 2019; Levesque & Quesnel-Vallée, 2019; Souto et al., 2021), the burden of proof should be on demonstrating that the finding is not a mere methodological or analytical artifact. I encourage both Lindström et al. and the wider research community preoccupied with the nexus of social capital and health outcomes to engage in further research to bring more datapoints and more clarity to this issue. To paraphrase Peter Lipton (2003), we need to perform an “inference to the best explanation” exercise and ask ourselves, abductively, “from what, if true, would this gendered disparity follow as a matter of fact?“. Quantitative methodological designs won't suffice. What is also sorely needed is to open the category of “gender” to analytical scrutiny and pluralistic conceptualizations.

## 3 Taking gender seriously

In Lindström et al.'s paper and almost all of cognate research, gender is a demographic and statistical variable. We should begin to listen more to our colleagues in social theory and gender studies and learn to see at least two more framings to it: gender as an ontological conundrum, and gender as an epistemological sensibility. Gender as an ontological conundrum is a conceptual umbrella to denote the long history of theorizing gender in the social sciences. To simplify a fascinatingly complex debate, there are theories that see gender not as a biological, natural, or “essence-like” entity but as a social construct through and through. This social constructionist school of thought approaches gender as a series of social performances that reproduce and reinforce particular constellations of ideologies (Butler, 2004). If we begin to look at Lindström et al.'s gendered statistics through this lens, and if we revisit the large literature on gender and social capital (e.g. Adkins, 2005; Healy et al., 2007; Karhina et al., 2019; Levesque & Quesnel-Vallée, 2019; O'Neill and Gidengil, 2013; Souto et al., 2021; Van Emmerik, 2006), we might be able to develop insights on ways in which the gendered disparity they have found could be ontologically real, rather than a mere methodological or analytical artifact. But even then, we encounter fundamental barriers coming from the nature of their data set: did the 2008 public health survey in Scania give only two choices for gender, thus reinforcing the currently criticized binary reductionism of gender to men/women only? If so, we need more fine-grained and “enlightened” quantitative data that parses the conceptual space of gender into multiple options that go well beyond the ideology of two genders only (see also Hyde et al., 2019; Johnston, 2016). Only when such data sets become available, will we be truly able to comprehend how gender plays out in the differential patterns of associations between social capital and mortality.

There is yet another framing of gender, namely gender as an epistemological sensibility, and it can act as a much-needed complement to the idea of gender as an ontological conundrum. Feminist epistemologies maintain that “differences in the social locations of inquirers make for epistemic differences” (Ashton & McKenna, 2020, p. 28) and thereby encourage researchers to explicitly account for their positionality and the situatedness of their knowledge claims (Simandan, 2016, 2002, 2019a-b, 2020). Instead of hiding one's identity behind statistics, impersonal language, and the rhetoric of neutrality, objectivity, and impartiality, feminist approaches to the production of scientific knowledge urge us to do the opposite and reflect on how the forms of social difference that we embody (race, ethnicity, age, gender, sexual orientation, social class, etc.) influence the choice of topics we research, the methods we use, and the manner in which we interpret the empirical evidence. Lindström et al.'s paper relies on a set of concepts such as tradition, trust, social capital, community, or gender, that carry an insufficiently acknowledged political and ideological load. My take-home message is that even the quantitatively oriented research that dominates *SSM - Population Health* must engage with this problematic, instead of just brushing it under the rug.

## Ethics statement

I declare no conflict of interest. The writing of this commentary was not funded from any source.
